# Analgesic effect of extracorporeal shock-wave therapy for frozen shoulder: A randomized controlled trial protocol

**DOI:** 10.1097/MD.0000000000021399

**Published:** 2020-07-31

**Authors:** Han-Yong Qiao, Li Xin, Shao-Lan Wu

**Affiliations:** a Department of Special Inspection; b Department of Pathology, The Third People's Hospital of Linyi, Shandong, 276023, China.

**Keywords:** extracorporeal shock-wave therapy, frozen shoulder, pain score, protocol

## Abstract

**Background:**

Till date only a few studies have reported the efficacy and clinical improvements obtained by extracorporeal shock-wave therapy (ESWT) on frozen shoulder. Limited by small number of studies and insufficient outcomes, it is important and necessary to conduct a new randomized controlled trial. The purpose of the present study is to determine whether ESWT could be more effective than oral steroid in treatment of frozen shoulder.

**Methods:**

This randomized, single-blind, superiority clinical trial was approved by the institutional review board in The Third People's Hospital of Linyi. The inclusion criteria were patients aged >18 years with shoulder pain and restriction in range of motion. A symptom duration >3 months was required, with no radiographic findings on anteroposterior shoulder plain radiographs except for osteoporosis. Group 1 patients were given 30 mg of oral prednisolone daily for 2 weeks as a single morning dose and then 15 mg daily for another 2 weeks. Group 2 patients received 3 sessions of ESWT on the first, 14th, and 28th days. The primary outcome measure was shoulder pain score. The secondary outcomes included Disabilities of the Arm, Shoulder, and Hand score, range of motion, satisfaction rate, and complications.

**Results:**

It was hypothesized that there would be a significant difference between ESWT and control groups in improving shoulder pain and functions in frozen shoulder.

**Trial registration:**

This study protocol was registered in Research Registry (researchregistry5736).

## 1 Introduction

Frozen shoulder is one of the common causes of shoulder pain and disability in the upper extremity. It affects the functions of glenohumeral joint, limiting both active and passive movements of the shoulder.^[1,2]^ The etiology of this disease is unknown but is associated with multiple factors, including female gender,^[3]^ diabetes,^[4,5]^ thyroid disease,^[6]^ trauma, stroke or myocardial infarction,^[5]^ or history of autoimmune diseases.^[7,8]^ The pathogenesis of frozen shoulder is poorly understood, which has limited the development of standard treatment protocols for the different stages of the disease.

Although there are a number of frozen shoulder treatment methods, including thermotherapy, psychrotherapy, electric percutaneous nerve treatment, ultrasound therapy, manual therapy, and taping therapy, 1 non-surgical treatment method that has been receiving attention recently is extracorporeal shock-wave therapy (ESWT).^[9–12]^ ESWT is a pulsed sound wave, characterized by short duration, high pressure amplitude, and relatively low tensile wave component. The mechanism of ESWT is not completely clear. However, it is speculated that ESWT may produce a reflexive analgesic effect by inducing excitability of the axon and destroying unmyelinated sensory fibers.^[13,14]^

ESWT has reported to treat a variety of pain conditions effectively and safely, including myofascial pain syndrome, knee pain, chronic pelvic pain syndrome, chronic rotator cuff tendonitis, sacroiliac joint pain, and frozen shoulder. Till date only a few studies have reported the efficacy and clinical improvements obtained by ESWT on frozen shoulder.^[15–18]^ Limited by small number of studies and insufficient outcomes, it is important and necessary to conduct a new randomized controlled trial. The purpose of the present study is to determine whether ESWT could be more effective than oral steroid in treatment of frozen shoulder. It was hypothesized that there would be a significant difference between ESWT and control groups in improving shoulder pain and functions in frozen shoulder.

## 2 Materials and methods

### 2.1 Study design

**Figure 1:**
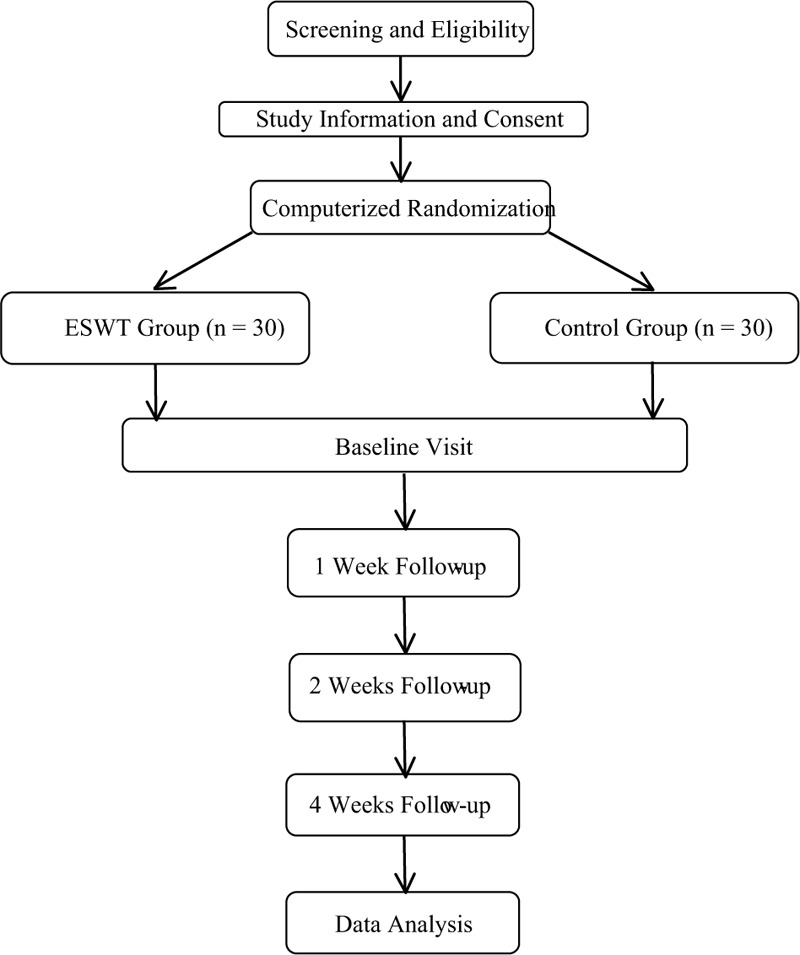
ESWT project flow chart.

This randomized, single-blind, superiority clinical trial was registered in Research Registry (researchregistry5736) and approved by the institutional review board in the third People's Hospital of Linyi (LYT082CM3804). The conduct of this study followed the Declaration of Helsinki principles and the reporting of this study adhered to the Consolidated Standards of Reporting Trials guidelines for randomized controlled trials. The flowchart of this trial is shown in Figure 1.

### 2.2 Participants

The inclusion criteria were patients aged >18 years with shoulder pain and restriction in range of motion. A symptom duration >3 months was required, with no radiographic findings on anteroposterior shoulder plain radiographs except for osteoporosis. No medical treatment, other than analgesics, was prescribed within the past 3 months. Patients were excluded from the study if they were pregnant, if they had had surgical intervention on the affected shoulder, if there was extensive scar around the shoulder, rotator cuff calcification, joint infection, lack of stability, rheumatoid arthritis or full thickness tear of shoulder rotator cuff, cervical radiculopathy or damage to the spinal cord, or history of cortisone injection in the affected area in the previous 6 weeks, or if they had other contraindications to shock wave treatment, including artificial pacemaker, use of anti-blood clotting medications, known bleeding disorder, known malignancy in the area intended for treatment, or epilepsy.

### 2.3 Randomization

Randomization was done by a secretary using a computer-generated randomization list in a 1:1 ratio with 30 numbers in each block. Every participant received a consecutive study number from 1 to 60 and received the treatment assigned according to the randomization list. All clinical personnel and outcome assessors were blinded to the intervention.

### 2.4 Intervention and control group

Group 1 patients were given 30 mg of oral prednisolone daily for 2 weeks as a single morning dose and then 15 mg daily for another 2 weeks. Group 2 patients received shock wave therapy using radial ESWT. Treatment of the affected tissue region was achieved by a sequence of 2000 shock wave pulses fired with a repetition frequency of 2 pulses per second. Energy level or intensity was set at a tolerable level by patient (0.2 mJ/mm^2^). The entire treatment lasted 15 min per session and was usually performed without local anesthetic drugs. All subjects received 3 sessions of ESWT on the first, 14th, and 28th days.

The participants were instructed to refrain from using any other conservative treatment, including physical therapy during their participation in this study. Patients were also discouraged to use non-steroidal anti-inflammatory medications during the following 2 weeks because of their inhibitory effects on recovery process. Acetaminophen 500 mg was ordered for pain in this period.

### 2.5 Outcome measures

All outcome measures were taken at baseline (before treatment) and at 4, 8, and 12 weeks from baseline. All assessments were performed by the same examiner who was blinded to the patients’ treatment groups. The primary outcome measure was shoulder pain score. Pain intensity was quantified using a 10-cm visual analog scale. Pain intensity was referred as 0 to 10, in which 0 = no pain at all and 10 = the worst pain possible. The secondary outcomes included Disabilities of the Arm, Shoulder, and Hand (DASH) score, range of motion (ROM), satisfaction rate, and complications. The DASH questionnaire was a 30-item questionnaire that measures the ability of a patient to perform certain upper extremity tasks. The scores obtained provide disability status and changes in symptoms and function over a period. Passive ROM of the affected shoulder was measured using a goniometer while the patient was sitting upright on a stool. Abduction, flexion, and external rotation ROMs were measured.

### 2.6 Sample size

The sample size calculation was based on a pilot study that we conducted on 18 patients (whose data were not included in the present study). In this prior study, the mean difference and standard deviation of the visual analog scale score at 30 days between the groups were 0.84 and 0.35, respectively. From this, it was determined that 20 subjects would be required to reach an α value of 0.05 and a power of 90%. It was estimated that the attrition rate due to reasons of late patient ineligibility could be up to 20% and, therefore, to account for this, the final sample size selected was n = 60 (30 per group).

### 2.7 Statistical analysis

Statistical calculations were performed using SPSS 20.0 software. For all comparisons, an alpha level of 0.05 was chosen to represent significance. Comparisons of means between groups were performed with a *t* test or Wilcoxon test, depending on the data distribution. Proportional comparisons were performed with the chi-square test. Mixed-model analysis of variance was used to investigate the simultaneous effects of time and rehabilitation on specific outcomes. Post hoc tests used the Tukey–Kramer correction to adjust for multiple comparisons.

## 3 Discussion

Frozen shoulder causes severe pain, restricts joints’ range of motion, and disturbs sleep when the pain is severe, all of which disrupt patients’ daily lives. Recently, ESWT was presented as a new way to treat frozen shoulder.

In the present study, we are conducting a protocol to figure out the effects of ESWT on frozen shoulder patients’ pain and functions. It was hypothesized that there would be a significant difference between ESWT and control groups in improving shoulder pain and functions in frozen shoulder. The main limitation of the current study was the inability to blind both the participants and the physicians in comparisons between ESWT and control group. This lack of blindness may have introduced some risk of bias from both the patients and the physicians. The outcome assessments from the adjudicators and all the statistical analyses were conducted in a blinded manner.

## Author contributions

**Conceptualization:** Shao-Lan Wu.

**Data curation:** Li Xin.

**Formal analysis:** Han-Yong Qiao, Li Xin.

**Funding acquisition:** Shao-Lan Wu.

**Investigation:** Han-Yong Qiao, Li Xin.

**Methodology:** Han-Yong Qiao, Li Xin.

**Resources:** Shao-Lan Wu.

**Software:** Li Xin.

**Supervision:** Shao-Lan Wu.

**Validation:** Han-Yong Qiao.

**Visualization:** Han-Yong Qiao.

**Writing – original draft:** Han-Yong Qiao.

**Writing – review & editing:** Shao-Lan Wu.
